# Longitudinal Course of Sex Steroids From Pregnancy to Postpartum

**DOI:** 10.1210/endocr/bqad108

**Published:** 2023-07-14

**Authors:** Jelena Dukic, Ulrike Ehlert

**Affiliations:** Department of Clinical Psychology and Psychotherapy, University of Zurich, 8050 Zurich, Switzerland; Department of Clinical Psychology and Psychotherapy, University of Zurich, 8050 Zurich, Switzerland

**Keywords:** sex steroids, hormones, pregnancy, postpartum, peripartum

## Abstract

**Context:**

Sex steroids (SS) typically rise during pregnancy and decline after birth, but no consistent reference values exist for these hormonal courses. We aimed to establish an overview of SS secretion patterns during the peripartum and to better understand how SS contribute to maternal and fetal pathologies.

**Evidence acquisition:**

A systematic literature search was conducted in accordance with the PRISMA guidelines using PubMed, Cochrane Library, and PsycINFO. Additionally, we conducted a supplementary manual search of references. Observational studies published in English and assessing estradiol, progesterone, and testosterone over the course of the peripartum in physically healthy female subjects were included, without restrictions on year of publication. Extracted data were analyzed descriptively and visually.

**Evidence synthesis:**

SS increase progressively during pregnancy, with an extremely wide range of reported concentrations, especially in the third trimester. In fact, reported concentrations varied up to 5000-fold at comparable measurement time points.

**Conclusions:**

A comprehensive understanding of the influence of SS levels on associated maternal and fetal pathologies is currently hindered by 2 main factors. First, reported SS levels vary widely during the peripartum period. Second, the current state of knowledge on how SS are associated with pathologies in mothers and babies is largely based on correlational studies, and causality thus remains unclear. Consequently, we recommend the development of a systematic reference framework that follows the suggestions presented in this review. This would enable the establishment of SS reference values for a healthy population, resulting in the possibility to draw conclusions about deviations and related pathologies.

The peripartum is accompanied by distinct hormonal changes in women. Sex steroids (SS) play a crucial role in the development and course of the peripartum. 17β-Estradiol (E2) and progesterone (P4) strongly increase during pregnancy, before dropping sharply after parturition (1-4). These endocrine changes are adaptive and ensure the maintenance of a healthy pregnancy and postpartum period (5). SS are involved in the control of a wide range of maternal and placental functions, as well as in the development of fetal organs during pregnancy. They are further essential for the induction of labor, maternal behavior after delivery, and lactation (6).

A physiological SS increase during pregnancy and a decrease postpartum are thus important for both the mother and the offspring (7). Deviations from this characteristic pattern can lead to a variety of pathologies and complications (8). For example, fetal intrauterine growth restrictions or a reduced birth weight or length of the newborn appear to be associated with deviating SS levels (9, 10). Regarding the later development of the offspring, excessively rising SS levels during pregnancy have been linked to an increased susceptibility to autism spectrum disorder (11, 12). Deviating SS concentrations during the peripartum may also be associated with physical and cognitive impairments in mothers, including preeclampsia, gestational diabetes, changes in the activity of maternal autoimmune diseases, deficits in working memory and cognitive performance, structural changes in the brain, and the later development of breast cancer (13-22). Moreover, changes in SS during the peripartum period have been related to psychological symptoms and disorders. Mood disorders, depressive symptoms, and restless legs syndrome in the peripartum, as well as anxiety symptoms, “baby blues,” and postpartum depression (PPD) in the postpartum period, are associated with SS values deviating from the norm (23-31). To better understand how deviations of SS in the peripartum can lead to the associated developmental, obstetric, autoimmune, neuronal, affective, and sleep pathologies and complications mentioned above, it is important to have reference values from healthy women for SS courses in the peripartum and to uncover the mechanisms of action that affect SS levels.

SS are controlled by the hypothalamic-pituitary-gonadal axis and are released in varying concentrations in both sexes. E2 and P4 are the major female sex hormones and testosterone (T) is the major male sex hormone. Both E2 and P4 are mainly produced by the ovaries in females (32). In addition to their endocrine and paracrine function, SS can also act as neuroactive steroids in the brain (33). E2, P4, and T are also produced in the brain, either through de novo synthesis from cholesterol or through resynthesis of local steroid metabolites (34). These neurosteroids can rapidly modulate neuronal excitability and functions, brain plasticity, and behavior (35). Additionally, a relationship between E2 and the serotonergic neurotransmission system may be involved in the neurobiological mechanisms of depression in women. E2 modulates the expression of genes that regulate serotonin neurotransmission and are implicated in the etiology of depression (36, 37). According to the hormone sensitivity hypothesis, some women seem to have an increased sensitivity to E2 fluctuations during reproductive transition phases (38, 39). Epigenetic mechanisms, such as DNA methylation patterns of the HP1BP3, TTC9B, and OXTR genes, may be responsible for this E2 sensitivity and the subsequent development of PPD (40, 41). Furthermore, it is assumed that SS affect receptors and genes that are involved in neuronal development and connectivity of the fetal brain, and this might therefore explain developmental disorders of the offspring (42). During pregnancy, the placenta regulates the maternal and fetal physiology by triggering the maternal and fetal hypothalamic-pituitary-gonadal axis (43). To better understand the etiopathology and direction of action between SS and the associated pathologies, longitudinal trajectories of these hormone release patterns, and their influencing factors should be considered.

To date, there is a lack of consistent evidence on potential factors influencing the measurement of SS during the peripartum period. Despite the widespread measurement and interpretation of SS in research and clinical decision making, important methodological issues that complicate an accurate measurement and interpretation of SS values are often overlooked. During pregnancy, maternal SS levels are reportedly influenced by maternal age, parity, body mass index (BMI), ethnicity, gender of the fetus, and lifestyle factors such as smoking (44-48). To our knowledge, there are currently no data on influencing factors specifically referring to the postpartum period, although general determinants of steroid hormone variability have been established. Age is considered the strongest predictor of SS concentrations, followed by BMI and race (49). Methodologically, SS are usually measured in blood serum or plasma. However, saliva sampling is viewed as an attractive alternative to blood sampling due to its practicality, noninvasiveness, and repeatability (50, 51), although concentrations should only be considered in relation to the respective specimen, and concentrations from different specimens should not be considered together (51-54). Moreover, reported SS concentrations are expected to vary depending on the assay method applied (54, 55), and even within the same type of assay, divergent results have been reported with regard to analytical accuracy and detection limits (56).

In sum, deviations from the physiological SS course in the peripartum are associated with different pathologies and complications in both mothers and their infants. At the same time, SS show numerous mechanisms of action through different biochemical pathways. To better understand the etiopathological significance of SS for peripartum symptoms and impairments, longitudinal analyses of SS over the entire period from pregnancy to postpartum should be considered. Furthermore, the evaluation of SS measurements in the peripartum should take into account general and pregnancy-specific maternal variables as well as methodological aspects such as the sample examined and the test procedure used. Consequently, it is essential to review all existing results from the literature on this topic. The present review aims to establish an overview of SS secretion patterns during the peripartum in physically healthy women while considering influencing maternal variables and methodological particularities. The overarching goal is to ultimately gain a better understanding of how deviations can lead to the associated complications and pathologies in both mothers and infants.

## Methods

The protocol for this systematic review was registered with the International Prospective Register of Systematic Reviews (PROSPERO; registration no. CRD42022301732). The systematic literature search and review were performed according to the Preferred Reporting Items for Systematic Reviews and Meta-Analyses (PRISMA) guidelines (57). All data were obtained from previously published studies; therefore, institutional review board approval was not required or obtained.

### Search Strategy and Data Sources

**Figure 1.**
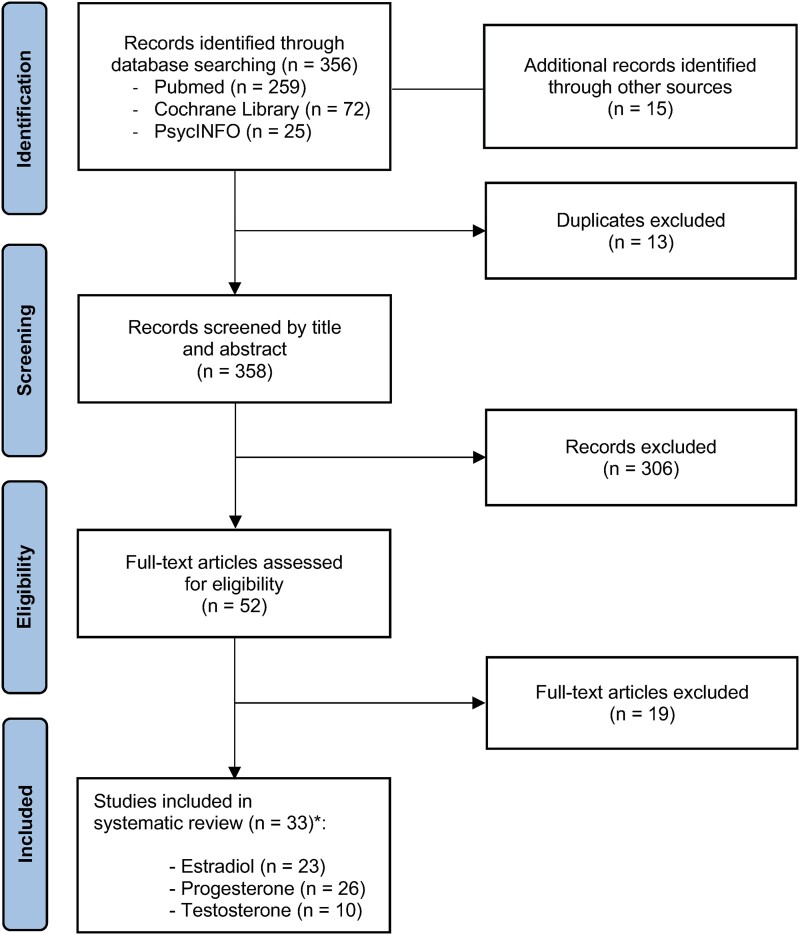
PRISMA flowchart of study selection for inclusion in the systematic review.

PubMed, Cochrane Library, and PsycINFO were searched on May 17, 2023, to identify relevant articles. Studies were eligible for inclusion if they were original research performed in humans, had an observational study design, and reported a SS concentration longitudinally no less than once during pregnancy and once during postpartum in pregnant and postpartum women (Fig. 1). Qualitative studies were not included in this review. There were no restrictions on year of publication. Results were limited to publications in the English language reporting human studies with physically healthy female subjects only. In articles reporting data from both a diseased sample and healthy controls, only data from healthy subjects were extracted. Only primary research articles were considered; review articles, reprint requests to editors, and comments were excluded from the final analysis. The main outcome measures were E2, P4, and T concentrations over the course of pregnancy and postpartum. Additionally, data on age, BMI, ethnicity/race, parity, specimen assessed, and method of analysis were obtained when possible.

A search strategy was developed using Medical Subject Headings (MeSH) and key words. The following MeSH terms and key words were applied for the search: *Gonadal Steroid Hormones* [MeSH], *Estradiol* [MeSH], *Estradiol Congeners* [MeSH], *Progesterone* [MeSH], *Progesterone Congeners* [MeSH], *Testosterone* [MeSH], *Testosterone Congeners* [MeSH], *Pregnancy* [MeSH], *Postpartum Period* [MeSH], *Peripartum Period* [MeSH], *Longitudinal Studies* [MeSH], *sex steroid(s)*, *sex hormone(s)*, *gonadal steroid(s)*, *steroid hormone(s)*, *reproductive hormone(s)*, *estradiol*, *estradiol concentration(s)*, *progesterone*, *progesterone concentration(s)*, *testosterone*, *testosterone concentration(s)*, *hormonal change(s)*, *hormonal fluctuation(s)*, *pregnancy/-ies*, *postpartum*, *peripartum*, *puerperium*, *longitudinal*, *course(s)*, *trajectory/-ies*.

A supplementary manual search of references was conducted in order to screen for additional publications. Titles and abstracts were reviewed independently by 2 reviewers to identify pertinent articles. Any disagreements were resolved by consensus.

### Data Extraction and Synthesis

Data regarding assessed SS, concentration of SS, measurement time points, method of SS analysis, number of participants in the respective study, maternal characteristics, and sample type were collected in an Excel spreadsheet. If a study was reported in multiple publications, these publications were pooled together under a single study ID. If further data were required or available data needed to be clarified, we attempted to contact the authors. Data were categorized for each SS separately and additionally in intervals of 4 weeks for the time of pregnancy. Concentrations that were not reported in [pg/mL] were converted into [pg/mL] using the University of Zurich (UZH) Unit Converter.

## Results

The systematic search identified a total of 356 articles. A further 15 articles were found by reviewing reference lists of screened articles, resulting in a total of 371 articles. After exclusion of duplicates, 358 records remained. Titles and abstracts of these records were screened, resulting in the additional exclusion of 306 records that did not meet the inclusion criteria for this review. For the remaining 52 publications, the full texts were critically appraised, and 19 were excluded because they did not report the outcome of interest. Finally, 33 articles were included in the systematic review and were classified into 3 groups: studies reporting E2 concentrations (n = 23), studies reporting P4 concentrations (n = 26), and studies reporting T concentrations (n = 10). Twenty-one reports were classified into more than one group. Figure 1 summarizes the steps involved in the literature selection based on the PRISMA guidelines. The final study group for assessment consisted of longitudinal, prospective observational studies published between 1971 and 2022. All studies relevant to this systematic review are summarized in Table 1 (14, 23, 25, 27, 28, 58-85).

**Table 1.** Overview of the reviewed studies

ID	Authors, year of publication, country of study origin, reference	Sample description	Parity	Measurement points in gestational weeks	Measurement points postpartum	Assessed hormones	Specimen	Method of analysis	Study topic
1	Molinet Coll et al, 2022, Spain (58)	n = 109 ⌀ Age 31.2 ⌀ BMI 25.1 Ethnicity/race n.a.	100% nulliparous	13, 34	45 days	E2 P4 Relaxin	Serum	E2: CLIA P4: CLEIA	Hormonal influence in stress urinary incontinence in the peripartum
	Parés et al, 2021, Spain, (59)	n = 109 ⌀ Age 31.2 ⌀ BMI 25.1 Ethnicity/race n.a.	100% nulliparous	13, 34	45 days	E2 P4 Relaxin	Serum	E2: CLIA P4: CLEIA	Hormonal changes and hemorrhoidal disease in the peripartum
2	Odogwu et al, 2021, Nigeria, (60)	n = 38 ⌀ Age 32.5 ⌀ BMI 21.3 Ethnicity/race African	34% nulliparous	17-21, 27-31, 36-41	6 weeks	E2 P4	Plasma	ELISA	Vaginal microbiome and hormonal changes in the peripartum
3	Kikuchi et al, 2021, Japan, (61)	n = 204 ⌀ Age 30.2 ⌀ BMI n.a. Ethnicity/race n.a.	48% nulliparous	13-21 (⌀15), 24-27 (⌀27)	0-6 days (⌀ 3 days)	E2 P4 T	Serum	ECLIA	Prepartal steroid production and PP depressive symptoms
4	Young et al, 2018, USA, (62)	n = 27 ⌀ Age 29.7 ⌀ BMI n.a. Ethnicity/race 82% White 15% Hispanic 3% Mixed	⌀ 1.8 parity	36	Within 4 days after delivery, 5-7 days, 21-27 days	E2 P4 T (+ further hormones)	Saliva	LC-MS/MS	Effects of placentophagy on maternal hormones
5	Bos et al, 2018, Netherlands, (63)	n = 86*^a^* ⌀ Age 31.6 ⌀ BMI n.a. Ethnicity/race n.a.	85% nulliparous	34	6 weeks	T COR	Saliva	EIA	Perinatal cortisol, T and caregiving quality
6	Voegtline, Costigan & DiPietro, 2017, USA, (64)	n = 158 ⌀ Age 32.1 ⌀ BMI n.a. Ethnicity/race 75% White 17% Asian 8% African-American	56% nulliparous	18, 24, 30, 36	6 months	T	Saliva	ELISA	Maternal T in pregnancy and fetal neuromaturation
7	Raga et al, 2016, Spain, (65)	n = 117 ⌀ Age 33.2 ⌀ BMI n.a. Ethnicity/race n.a.	n.a.	32-35	6 weeks	P4	Plasma	CLEIA	Periodontal parameters and P4 in the peripartum
8	Deligiannidis et al, 2016, USA, (66)	n = 56 ⌀ Age 31.9 ⌀ BMI n.a. Ethnicity/race 85.7% Caucasian	44% nulliparous	27-34, 33-38	1-5 days, 2-9 weeks	P4 GABA DOC PREG	Plasma	LC-MS/MS	Neuroactive steroid and γ-aminobutyric acid profiles in women at risk of PPD
9	Enninga et al, 2015, USA, (67)	n = 38 ⌀ Age 28.2 ⌀ BMI n.a. Ethnicity/race n.a.	100% nulliparous	≤ 8, 9-12, 13-17, 18-22, 23-27, 28-32, 33-37, 38-40, 41-42	6 weeks	E2 P4 Estrone PRL CRH	Plasma	ELISA	Fetal sex-based differences in maternal hormones, angiogenic factors, and immune mediators in the peripartum
10	Mehta et al, 2014, USA, (68)	n = 62 ⌀ Age 34.1 ⌀ BMI 29.4 Ethnicity/race 87% White Non-Hispanic 13% Other	⌀ 0.8 parity	2.7-12.0 (⌀ 7.7 GW), 24.4-38.9 (⌀ 34.3 GW)	1.3-11.9 weeks (⌀ 4.9 weeks)	E2 Estriol	Plasma	IA	Early predictive biomarkers of PPD
11	Farrar et al, 2014, UK, (69)	n = 23*^a^* ⌀ Age 30 ⌀ BMI 26.5 Ethnicity/race n.a.	0% nulliparous	During the final 2 weeks of each trimester	12 weeks	E2 P4 COR PRL SHBG	Plasma	RIA	Assessment of cognitive function across pregnancy in relation to hormonal changes
12	Akhter et al, 2013, Sweden, (70)	n = 57 ⌀ Age 30 ⌀ BMI 24 Ethnicity/race n.a.	51% nulliparous	13, 23, 37	1 year	E2	Serum	ECLIA	Hormones and artery wall layer dimensions during normal pregnancy
13	Henry & Sherwin, 2012, Canada, (71)	n = 55*^a^* ⌀ Age 31.4 ⌀ BMI n.a. Ethnicity/race n.a.	100% nulliparous	34-38	12 weeks	E2 P4 T COR	Serum	CLIA	Hormones and cognitive functioning during late pregnancy and PP
14	O'Keane et al, 2011, UK, (72)	n = 70 ⌀ Age 33.1 ⌀ BMI n.a. Ethnicity/race n.a.	63% nulliparous	36	1-6 days	P4 COR CRH ACTH Oestriol	n.a. (not specified whether serum or plasma)	CLIA	Maternal HPA axis during the early PP and the PP “blues’
15	Chatzicharalampous et al, 2010, Greece, (73)	n = 57 ⌀ Age 32.7 ⌀ BMI 29.6 Ethnicity/race n.a.	49% nulliparous	At admission to delivery	1 day, 2 days, 3 days, 4 days	E2 P4 T	Serum	n.a.	Reproductive hormones and PP mood disturbances
16	Figuero et al, 2010, Spain, (74)	n = 42*^a^* ⌀ Age n.a. ⌀ BMI n.a. Ethnicity/race n.a.	n.a.	12-14, 23-25, 33-36	3 months	E2 P4	Saliva	ELISA	The influence of hormonal variations on gingival changes in the peripartum
	Carrillo-de-Albornoz et al, 2010, Spain, (75)	n = 48*^a^* ⌀ Age n.a. ⌀ BMI n.a. Ethnicity/race n.a.	n.a.	12-14, 23-25, 33-36	3 months	E2 P4	Saliva	ELISA	The influence of hormonal variations on the subgingival biofilm in the peripartum
17	Dzaja et al, 2009 Germany	n = 9*^a^* ⌀ Age 32.9 ⌀ BMI n.a. Ethnicity/race n.a.	n.a.	36	12 weeks	E2 P4 T COR PRL	Plasma	ECLIA	E2 levels in women with and without restless legs syndrome in the peripartum
18	Soldin et al, 2005, Sweden, (76)	n = 50 ⌀ Age 30 ⌀ BMI n.a. Ethnicity/race 100% Caucasian descent/Non-Hispanic	100% nulliparous	12, 22, 32	1 year	E2 P4 COR A4 DHEA DHEA-S	Serum	LC-MS/MS	Steroid hormone levels in the peripartum
19	LeResche et al, 2005, USA, (77)	n = 35 ⌀ Age 28.5 ⌀ BMI n.a. Ethnicity/race 85.7% White	43% nulliparous	12, 24, 37	1 year	E2 P4	Saliva	ELISA	Temporomandibular disorders and hormonal changes in the peripartum
20	Marnach et al, 2003, USA, (78)	n = 46 ⌀ Age 28 ⌀ BMI n.a. Ethnicity/race 94% White 6% Asian	⌀ 0.7 parity	8-12, 16-22, 34-36	5-6 weeks	E2 P4 COR Relaxin	Serum	E2: RIA P4: ELISA	The relationship between joint laxity and maternal hormones during peripartum
21	Serin et al, 2001, Turkey, (14)	n = 20*^a^* ⌀ Age 23.1 ⌀ BMI n.a. Ethnicity/race n.a.	n.a.	28-32	6 weeks	E2 T DHEA-S SHBG A4	Serum	RIA	Comparison of hormonal levels of preeclamptic patients and healthy controls during peripartum
22	Hohlagschwandtner et al, 2001, Austria, (28)	n = 193 ⌀ Age 28.2 ⌀ BMI n.a. Ethnicity/race n.a.	⌀ 1.5 parity	38-40	1 day, 3 days	T COR PRL E2*^b^* P4*^b^*	Serum	RIA	Correlation between T levels and peripartal mood states
23	Elenkov et al, 2001, USA, (79)	n = 18*^a^* ⌀ Age n.a. ⌀ BMI n.a. Ethnicity/race n.a.	n.a.	33-36	3-6 weeks	E2 P4 COR EPI NE	Plasma	RIA	Biomarkers of autoimmune disease activity in the peripartum
24	Lee, McEnany & Zaffke, 2000, USA, (80)	n = 31*^a^* ⌀ Age 31.6 ⌀ BMI n.a. Ethnicity/race n.a	n.a.	11-12, 23-24, 35-36	3-4 weeks, 11-12 weeks	P4	Serum	n.a.	REM sleep, hormonal changes and mood states in peripartal women
25	Buckwalter et al, 1999, USA, (25)	n = 19 ⌀ Age 33.1 ⌀ BMI n.a. Ethnicity/race n.a	n.a.	⌀ 19.8 days prior to delivery	⌀ 26.5 days	E2 P4 T COR DHEA	Serum	RIA	Effects of steroid hormones on cognition and mood in the peripartum
26	Alvarez et al, 1995, Spain, (81)	n = 25 ⌀ Age 29.5 ⌀ BMI 22.6 Ethnicity/race n.a	n.a.	9-12, 21-24, 32-35	2-4 days, (post lactation)*^c^*	E2 P4 PRL	Plasma	RIA	Lipoproteins, post-heparin lipases and hormones in the peripartum
27	Asher et al, 1995, Israel, (82)	n = 25 ⌀ Age 28.8 ⌀ BMI n.a. Ethnicity/race n.a	n.a.	Just before delivery	3 days	P4 Estrogen PRL LH FSH TSH	Plasma	RIA	Mood and hormonal changes in the peripartum
28	Montelongo et al, 1992, Spain, (83)	n = 12*^a^* ⌀ Age 28.7 ⌀ BMI 22.4 Ethnicity/race n.a	n.a.	9-10, 21-23, 32-34	2-4 weeks (post lactation)*^c^*	E2 P4 PRL	Plasma	RIA	Lipoproteins and hormones in the peripartum in healthy and diabetic women
29	O'Hara et al, 1991, USA, (23)	n = 182 ⌀ Age 27 ⌀ BMI n.a. Ethnicity/race n.a	57% nulliparous	34, 36, 38	1 day, 2 days, 3 days, 4 days, 6 days 8 days	E2 P4 COR PRL Estriol	Serum	ELISA	PP mood disorders and psychological, environmental, and hormonal variables
30	Meulenberg & Hofman, 1989, Netherlands, (84)	n = 36 ⌀ Age n.a. ⌀ BMI n.a. Ethnicity/race n.a	n.a.	14-19, (20-26, 27-34)*^b^*, 35-40	6 weeks	P4	Plasma, saliva	RIA	Correlation of salivary P4 and plasma P4 in the peripartum
31	Ances, Hisley & Haskins, 1971 USA, (85)	n = 14 ⌀ Age 26 ⌀ BMI n.a. Ethnicity/race n.a	n.a.	Prior to labor	1h	P4	Plasma	RIA	P4 levels throughout the course of labor

Note: Means are represented by ⌀.

Abbreviations: A4, 4-androstenedione; ACTH, adrenocorticotropic hormone; BMI, body mass index; CLEIA, competitive chemiluminescent enzyme immunoassay; CLIA, chemiluminescent immunoassay; COR, cortisol; CRH, corticotropin-releasing hormone; DHEA, dehydroepiandrosterone; DHEA-S, dehydroepiandrosterone sulfate; DOC, deoxycorticosterone; E2, 17β-estradiol; ECLIA, electrochemiluminescence immunoassay; EIA, enzyme immunoassay; ELISA, enzyme-linked immunosorbent assay; EPI, epinephrine; FSH, follicle-stimulating hormone; GABA, gamma-aminobutyric acid; HPA, hypothalamic-pituitary-adrenal; IA, immunoassay; LC-MS/MS, liquid chromatography with tandem mass spectrometry; LH, luteinizing hormone; n.a., not available; NE, norepinephrine; P4, progesterone; PREG, pregnenolone; PRL, prolactin; PP, postpartum; PPD, postpartum depression; RIA, radioimmunoassay; SHBG, sex hormone binding globulin; T, testosterone; TSH, thyroid-stimulating hormone.

a Only physically healthy peripartal female subjects considered.

b Concentrations were not reported.

c Not considered for this overview.

### Sex Steroid Concentrations and Methods of Analysis

**Figure 2.**
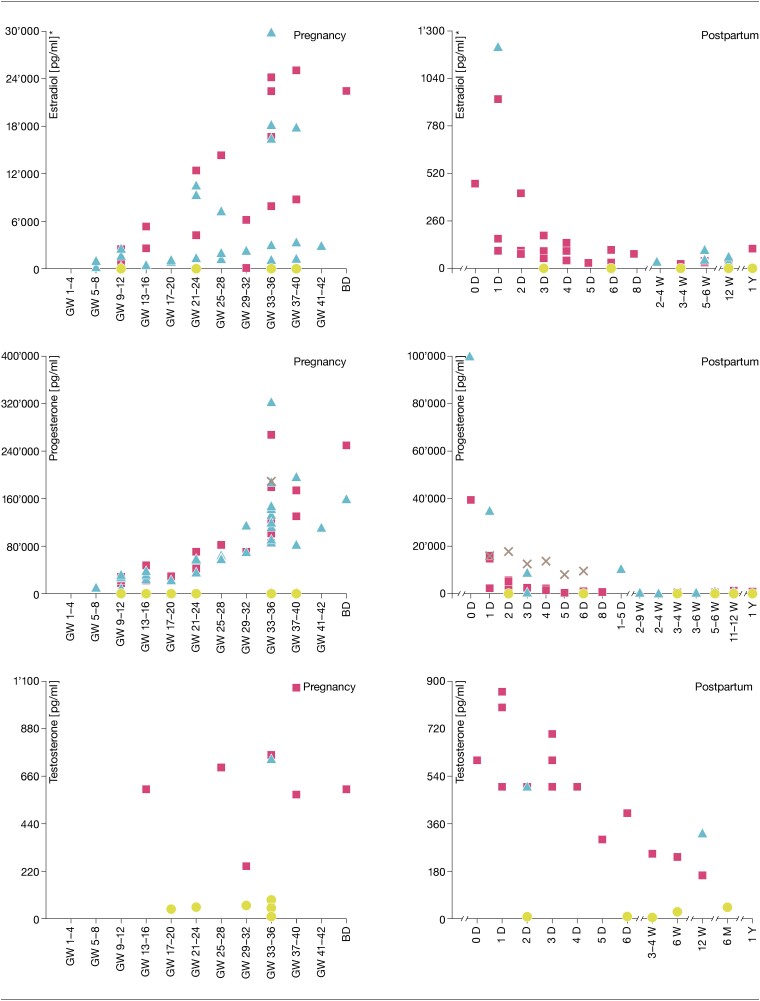
Sex steroid concentrations (E2, P4, T) measured longitudinally during pregnancy and postpartum in plasma, serum, and saliva.

Table 2 shows a summary of the reported values for the different SS and peripartum phases (14, 23, 25, 27, 28, 59-85). Precisely reported E2, P4, and T concentrations are presented in Supplementary Tables S1 to S6 (86). As can be seen in Fig. 2, over the course of the peripartum, E2 increases progressively during pregnancy, with a very wide range of reported concentrations in serum and plasma especially in the third trimester, even within the same specimen. After delivery, E2 drops dramatically and remains low from the first week postpartum onwards. P4 increases steadily during pregnancy, with the highest concentrations being reported around gestational weeks 33 to 36, but again with a very broad range of reported concentrations in serum and plasma. After delivery, P4 drops sharply and remains low from the second week postpartum onwards. T slightly increases during the second and third trimester of pregnancy and gradually falls after delivery, with the highest concentrations being reported in serum samples.

**Table 2.** Reported ranges of 17 β-estradiol, progesterone, and testosterone in the different phases of peripartum in different specimens (in pg/mL)

	17β-Estradiol range	Progesterone range	Testosterone range
First trimester in serum	870-2475*^a^*	17 500-28 400	Not reported
First trimester in plasma	165-2514	8640-31 476	Not reported
First trimester in saliva	1-6	143-250	Not reported
Second trimester in serum	2583-14 325*^a^*	22 473-82 100	600-700
Second trimester in plasma	436-10 474	535-64 149	Not reported
Second trimester in saliva	5-17	200-545	44-53
Third trimester in serum	92-25 070*^a^*	70 500-267 499	243-1070
Third trimester in plasma	1090-29 843*^b^*	1975-322 430	738*^c^*
Third trimester in saliva	24-75	56-1543	9-86
PP days 0-2 in serum	80-927	2300-39 600	500-860
PP days 0-2 in plasma	1211*^c^*	34 816-99 900	Not reported
PP days 0-2 in saliva	Not reported	106*^c^*	7*^d^*
PP days 3-8 in serum	31-180	500-2800	300-700
PP days 3-8 in plasma	55*^c^*	261-8710	Not reported
PP days 3-8 in saliva	0.2-0.5	10*^c^*	8*^c^*
PP weeks 2-12 in serum	24-41*^a^*	200-1263	164-246
PP weeks 2-12 in plasma	35-100*^b^*	26-870	323*^c^*
PP weeks 2-12 in saliva	0.02-0.2	11-30	4-26
PP 1 year in serum	<100-107.9	860*^c^*	Not reported
PP 1 year in plasma	Not reported	Not reported	Not reported
PP 1 year in saliva	0.5*^c^*	18*^c^*	Not reported

Abbreviation: PP, postpartum.

a After exclusion of one extreme outlier (78).

b After exclusion of one extreme outlier (79).

c Only reported once.

d Reported twice at the same level.

**Figure 3.**
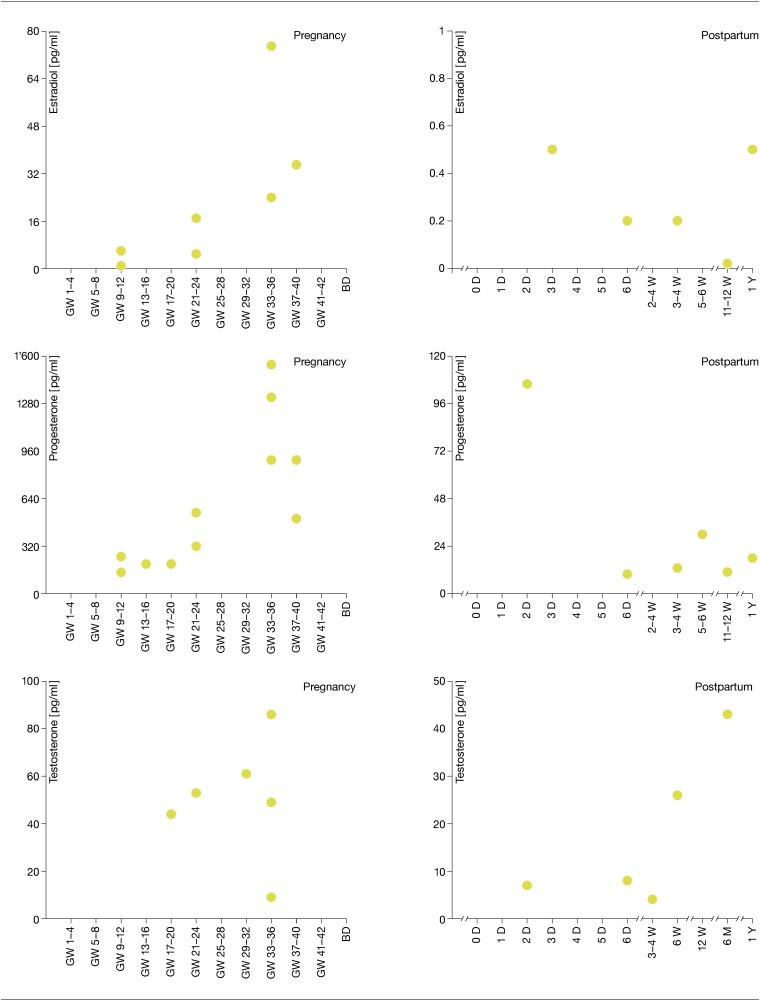
Sex steroid concentrations (E2, P4, T) measured longitudinally during pregnancy and postpartum in saliva only.

For all 3 hormones, reported salivary concentrations are significantly lower than concentrations in blood serum and plasma. Therefore, Fig. 3 presents the salivary concentrations again, separately on an adjusted scale (62-64, 74, 75, 77, 84). As can be seen here, even the saliva samples show large variations in the reported SS depending on the respective study, and again especially in the third trimester, but the variations are less dramatic than in the blood samples. Although SS were less frequently assessed in saliva during the postpartum period, a decrease was also observed for all 3 SS within the first days after birth. Thereafter, however, the assessed studies report increases in all 3 SS concentrations at about 5 to 6 weeks postpartum. After 6 months, T concentrations reached the same level as during pregnancy.

Table 1 depicts all methods of analysis used for the quantification of sex steroids in the assessed studies (14, 23, 25, 27, 28, 59-85). Radioimmunoassay (RIA) was used most frequently for serum and plasma samples while enzyme-linked immunosorbent assay (ELISA) was used most frequently for saliva samples.

### Maternal Variables

As shown in Table 1, the mean maternal age was reported in 28 of the included studies and ranged between 23.1 and 34.1 years. The mean maternal BMI was reported in 8 of the included studies and ranged between 21.3 and 29.6 kg/m^2^. Eight of the included studies reported either maternal ethnicity or race. The vast majority of assessed women across these studies were white/Caucasian. Parity was reported in 18 of the included studies, in which most assessed women were nulliparous. Maternal variables were generally reported as means in terms of demographic variables of the assessed samples but were not controlled for with regard to SS levels.

## Discussion

The aim of this paper was to provide an overview of existing research regarding the longitudinal analysis of SS in the peripartum period. The existing knowledge of a rise especially of E2 and P4 during pregnancy, followed by a strong decline directly after birth, was also reflected in the results of the 33 studies included in this review. However, the studies showed huge differences in the reported SS concentrations at comparable gestational ages. Especially during the third trimester, the values varied widely for the same SS, specimen, and collection times. For example, E2 measured in plasma ranges from 2967 to 14 659 506 pg/mL during the period of 33 to 36 weeks of gestation, which represents an almost 5000-fold difference for one SS in the same specimen and gestational period. Across all collection time points and hormones, the widest range of measured concentrations was observed during this period of 33 to 36 weeks of gestation, while at the same time the majority of the data were collected during this time period.

Our results confirm the findings of Kuijper et al (1), who reviewed studies reporting hormone concentrations in fetal (serum) and maternal systems (serum, amniotic fluid, saliva, and urine) and found very different SS concentrations at comparable gestational ages reported across the included studies, especially for estrogen concentrations. Our review demonstrates that this is true for P4 as well, not only during pregnancy but also in the first days after birth.

When interpreting these large differences in SS levels, it is important to consider some methodological aspects. Across all 3 SS, serum and plasma levels were found to be of similar magnitude whereas saliva levels were comparatively extremely low. The reviewed studies employed a variety of different methods of analysis. Immunoassay (IA) was most commonly used, with 30 of the 33 studies measuring SS using IA methods and only 3 studies applying mass spectrometry (MS). Radioimmunoassay (RIA) was the most common method for serum and plasma samples and ELISA was most common for saliva samples. RIA, ELISA, enzyme immunoassay (EIA), chemiluminescence enzyme immunoassay, chemiluminescence immunoassay (CLIA), and chemiluminescence enzyme immunoassay (CLEIA) are considered as IA methods, while liquid chromatography-tandem mass spectrometry (LC-MS/MS) is a MS assessment method (87, 88). For serum and plasma samples, MS-based methods have been shown to be more sensitive, specific, and reliable than IA methods. However, there are numerous drawbacks of MS, such as a high cost of the mass spectrometer, the need for highly trained professionals, and the availability of commercial reagents (89-93). At the same time, IAs are widely used for the quantification of steroid hormones due to their multiple advantages, including high throughput of samples, relatively simple and fast setup and execution, relatively low cost, and lack of need for highly qualified personnel (94, 95). It has also been shown that a well-validated IA is not inferior to MS (93). Therefore, most steroid measurements are still performed using IA methods (90), as demonstrated in the present review. However, the large variations in reported SS levels may reflect the poor sensitivity of IA methods, since these methods may give rise to inaccurate results due to cross-reactions (96) A further reason for the large differences between different studies arises from inter- and intraassay variance (54, 55). Divergent results have even been reported within the same specimen and using the same assessment method (56, 89). This was also found in the present review: For example, P4 assessed using RIA in serum samples at 33 to 36 gestational weeks ranges from 1975 to 187 278 pg/mL. Comparisons within MS were not possible in the reviewed studies, since MS was never applied multiple times within the same SS and specimen.

Besides the aforementioned analytical challenges, maternal factors such as age, BMI, race/ethnicity, or individual lifestyle may influence SS levels (44-48, 49). Although these variables are reported in the descriptive statistics of the included studies, they were rarely included as covariates or control variables in the statistical analyses. Therefore, their potential effect on SS levels cannot be estimated. It is well known, for instance, that older pregnant women tend to have lower E2 and T levels compared to younger women, and P and T levels are notably higher in African American than in Caucasian or Hispanic women (45, 46). Especially in the case of multiple influencing factors, such maternal factors could provide a potential explanation for the broad differences in SS values reported across the studies and should therefore be considered when evaluating SS values in the peripartum period.

Moreover, the reviewed studies often showed very large time intervals between successive assessment time points. To be able to describe the processes of adaptation from pregnancy to the postpartum, the last measurement before birth and the first measurement after birth are crucial. These 2 time points should be as close together as possible in order to observe the significant decline in hormone levels directly after birth. In addition, little is known about postpartum hormonal adaptation and how quickly SS levels return to nonpregnant baseline levels. Factors potentially influencing SS concentrations such as breastfeeding, sleep patterns, or BMI must also be taken into account in this regard.

Furthermore, little is known about intraindividual differences in hormonal courses during the peripartum. It is conceivable that not all women show an equally large increase in E2 and P4 and that the rate of hormonal decline after birth varies from woman to woman. It would therefore be important to establish whether different types of hormonal trajectories exist in the peripartum period. For example, there may be a group of women with consistently low SS levels and another with very high or varying levels, which may likewise serve to explain the strongly differing hormone levels reported.

As mentioned above, the present review aimed to provide an overview of existing research on the longitudinal analysis of SS in the peripartum period. We further wished to gain a better understanding of how SS deviations may be associated with complications and pathologies in both mothers and babies. Unfortunately, this latter objective could not be achieved, largely because to date, there are no clearly defined reference ranges for SS in the peripartum period. The consulted publications mostly do not clearly define when SS values are defined as deviant, and even when divergent ranges are defined, they are inconsistent across the studies. If one is to assume that certain complications and pathologies are associated with deviating SS values, the expected normal range of values must first be specified. Only then will it be possible to draw conclusions regarding deviations and associated pathologies. As the current state of knowledge is based mainly on correlational studies, which have found an association between SS levels and certain pathologies or complications, we cannot draw any causal conclusions about this relationship. Moreover, the understanding of this relationship is further limited by the fact that studies comparing symptomatic with asymptomatic groups have only found small differences in absolute SS levels and small effect sizes. A crucial first step for future research is to clarify the normal physiological range of SS during the peripartum period more precisely, enabling deviations from this range to be identified more clearly and related to pathologies and complications. Furthermore, it is important to consider that the relationship between fetal and maternal circulating hormone levels is not equal, and they should therefore not be equated (97, 98). While fetal circulating E2 and P4 levels are mainly of placental origin, maternal hormone levels arise from different sources (maternal pituitary and adrenal glands, liver, and placenta). Maternal and fetal blood are constantly exchanged, but hormones larger than 0.7 kDa are almost unable to pass the placenta from the maternal to the fetal compartment, and SS concentrations thus differ between maternal and fetal blood (9, 99, 100). Consequently, one should measure maternal SS levels when investigating maternal pathologies and complications, and fetal SS levels when investigating fetal pathologies and complications. As an example, potential associations with autism spectrum disorders in the offspring should be examined based on fetal SS from umbilical blood.

Some limitations must also be considered with respect to the present review. First, as we only included studies from which hormone data from pregnancy and postpartum were available, a selection bias is possible. Second, due to our focus on describing changes between pregnancy and puerperium in the same women, we did not include studies that examined SS values cross-sectionally. Third, we only used the SS levels of physically healthy subjects and did not compare them with the SS levels of subjects who were not in good physical health.

## Conclusions

We aimed to provide an overview of existing research on the longitudinal analysis of SS in the peripartum period. The reviewed studies revealed widely varying SS levels during the female reproductive transition phase of pregnancy to postpartum, with different authors reporting very different SS values at comparable time points. Methodological aspects should be considered when interpreting these large differences. To achieve reliable results, the choice of analytical method should be carefully weighed, and a distinction should be made between serum, plasma, and saliva samples. Besides methodological aspects, maternal characteristics are also highly relevant and should be addressed when interpreting SS levels in the peripartum. For a valid and reliable interpretation of SS concentrations in the peripartum, assessments should include different age groups and ethnicities, as well as different BMI classes, and should take into account the sex of the fetus. Furthermore, time intervals of SS sampling should ideally be as close together as possible in order to observe rapid changes and adaptive processes in these hormones. We further sought to gain a better understanding of how SS deviations may be associated with complications and pathologies in both mothers and their offspring. According to our findings, knowledge regarding the relationship between SS levels and potentially associated pathologies and complications is mainly based on correlational studies, and causality thus remains unclear. In future research, physically and mentally healthy mothers who have given birth to long-term healthy children should be examined throughout the whole course of pregnancy and postpartum. This would enable normal values in a healthy population to be derived, consequently allowing for conclusions regarding deviations and associated pathologies. In view of the present findings, we recommend the development of a systematic reference framework for clinicians and researchers alike, which follows the aforementioned suggestions. This should foster our understanding of how SS contribute to maternal and fetal pathologies and perhaps even how they might be prevented.

## Data Availability

Original data generated and analyzed during this study are included in this published article or in the data repositories listed in References.

## Abbreviations

BMI: body mass index
E2: 17β-estradiol
ELISA: enzyme-linked immunosorbent assay
IA: immunoassay
MS: mass spectrometry
P4: progesterone
PPD: postpartum depression
PRISMA: Preferred Reporting Items for Systematic Reviews and Meta-Analyses
RIA: radioimmunoassay
SS: sex steroids
T: testosterone
